# Cytokines shape chemotherapy-induced and ‘bystander' senescence

**DOI:** 10.18632/aging.100171

**Published:** 2010-07-16

**Authors:** Zdenek Hodny, Sona Hubackova, Jiri Bartek

**Affiliations:** ^1^ Department of Genome Integrity, Institute of Molecular Genetics, v.v.i., ASCR, CZ-142 20 Prague 4, Czech Republic; ^2^ Institute of Cancer Biology and Centre for Genotoxic Stress Research, Danish Cancer Society, DK-2100 Copenhagen, Denmark

The permanent proliferation arrest, distended morphology and other phenotypic features characteristic of cellular senescence can be triggered by telomere attrition (replicative senescence) and various stresses such as activated oncogenes or genotoxic treatments (premature senescence). Physiological relevance of cellular senescence is apparent from its emerging roles in aging, in tumor pathogenesis as an inducible barrier to tumor progression, and in response to radiotherapy and chemotherapy [1]. Mechanistically, senescence induction and maintenance involve the major tumor suppressor pathways of pRB and p53, and persistent signaling of the DNA damage response (DDR) machinery [1]. An integral part of DNA damage signaling and senescence is the activation of a complex cytokine network [1,2] including proinflammatory species IL-6 and IL-8 and reorganization/multiplication of a specific nuclear compartment, PML nuclear bodies (PML NBs) [1,3]. Despite the critical roles of promyelocytic leukemia protein (PML), the structural component of PML NBs, in PML NBs assembly, tumor suppression, and stabilization and activation of p53 after various stresses, the molecular basis of PML regulation and its interplay with the senescence-associated secretory cytokine network are not well understood. A new study [4] now sheds light on the involvement of PML NBs in cellular senescence evoked by a spectrum of genotoxic drugs including clinically used chemotherapeutics, and provides important mechanistic insights into regulation of PML expression, causal relationship with cytokine signaling, and surprising lack of dependence on p53.

Relevant findings preceding this study include the recent demonstration of cytokine signaling pathways involved in drug-evoked senescence [5], and the fact that chemotherapy-induced senescence can occur in neighboring cells through so-called ‘bystander' effects [6]. The new work by Hubackova and colleagues [4] now shows that exposure of human normal and cancer cells to genotoxic drugs including those used to treat human malignancies such as camptothecin and etoposide, at concentrations evoking senescence and achievable in tissues during chemotherapy, resulted in enhanced formation of PML NBs, elevated PML transcript levels and activated JAK/STAT signaling indicative of cytokine involvement. As both endogenous PML transcript levels and PML promoter-driven luciferase activity were suppressed by chemical inhibition or RNAi-mediated knock-down of JAK1 kinase, the data reveals a key role of JAK1-controlled signaling in PML transcription induced by genotoxic stress. Furthermore, in contrast to oncogene-induced senescence where PML expression is controlled by p53, the experiments of Hubackova et al. with both p53-negative cells and regulatable dominant-negative allele of p53 showed that JAK1-regulated transcription of PML in response to genotoxic drugs is p53-independent [4].

Considered within the context of other data in the field, these new results [4] help us better understand the interplay of PML with cytokine signaling in drug-induced and ‘bystander' senescence, phenomena highly relevant for aging, cancer biology and treatment response.

**Figure 1.**
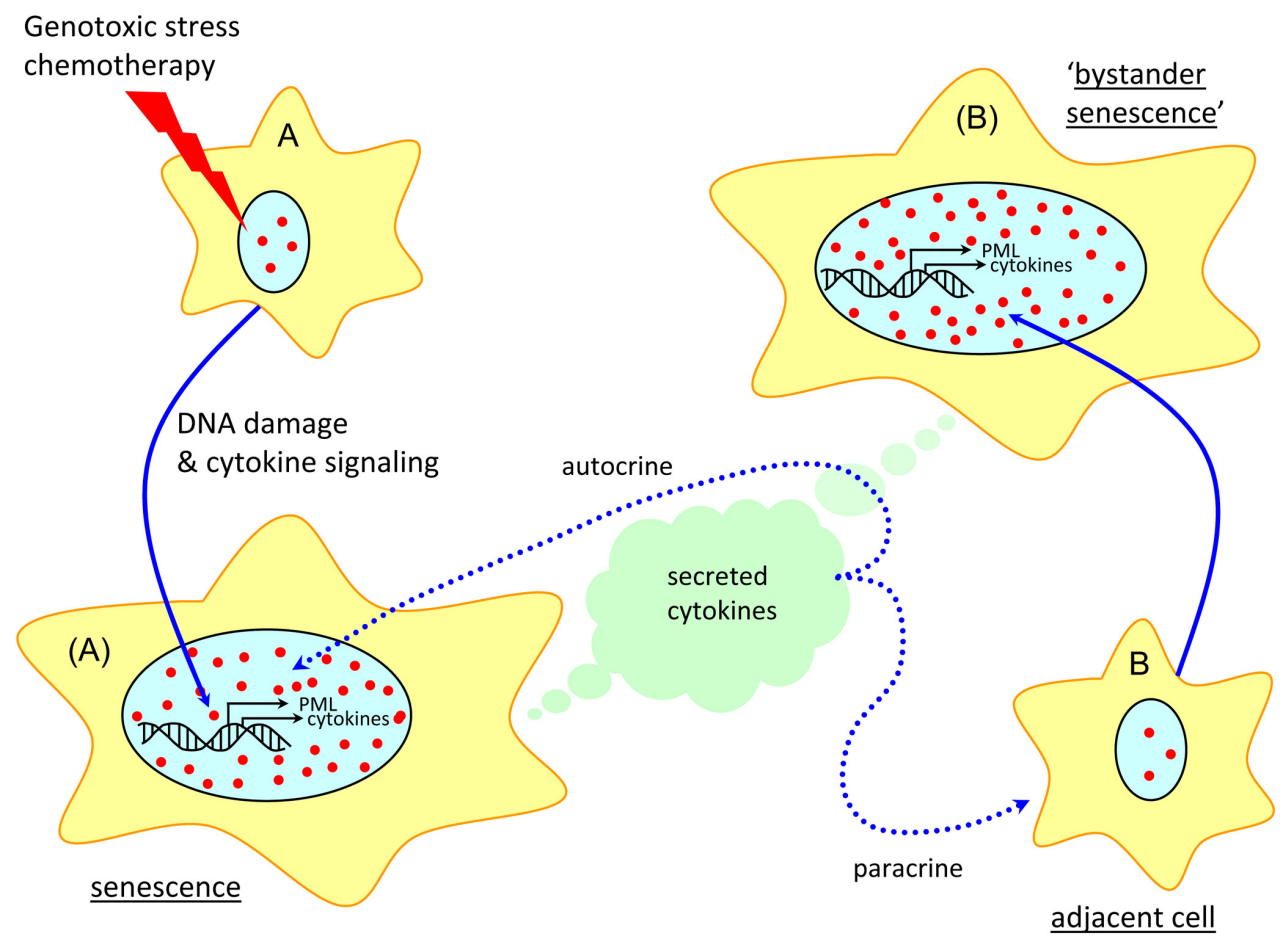
Model of PML and cytokine signaling in drug-induced and ‘bystander' senescence. Cytokine secretion and autocrine/paracrine signaling triggered by the DDR machinery upregulate PML expression and formation of PML NBs, collectively leading to cellular senescence, both directly and through ‘bystander' effects.
